# Novel resistance to Cydia pomonella granulovirus (CpGV) in codling moth shows autosomal and dominant inheritance and confers cross-resistance to different CpGV genome groups

**DOI:** 10.1371/journal.pone.0179157

**Published:** 2017-06-22

**Authors:** Annette J. Sauer, Eva Fritsch, Karin Undorf-Spahn, Petr Nguyen, Frantisek Marec, David G. Heckel, Johannes A. Jehle

**Affiliations:** 1 Institute for Biological Control, Julius Kühn Institute (JKI), Federal Research Centre for Cultivated Plants, Darmstadt, Germany; 2 Biology Centre of the Czech Academy of Sciences, Institute of Entomology, České Budějovice, Czech Republic; 3 University of South Bohemia, Faculty of Science, České Budějovice, Czech Republic; 4 Max Planck Institute for Chemical Ecology, Department of Entomology, Jena, Germany; Ecole des Mines d'Ales, FRANCE

## Abstract

Commercial Cydia pomonella granulovirus (CpGV) products have been successfully applied to control codling moth (CM) in organic and integrated fruit production for more than 30 years. Since 2005, resistance against the widely used isolate CpGV-M has been reported from different countries in Europe. The inheritance of this so-called type I resistance is dominant and linked to the Z chromosome. Recently, a second form (type II) of CpGV resistance in CM was reported from a field population (NRW-WE) in Germany. Type II resistance confers reduced susceptibility not only to CpGV-M but to most known CpGV isolates and it does not follow the previously described Z-linked inheritance of type I resistance. To further analyze type II resistance, two CM strains, termed CpR5M and CpR5S, were generated from parental NRW-WE by repeated mass crosses and selection using the two isolates CpGV-M and CpGV-S, respectively. Both CpR5M and CpR5S were considered to be genetically homogeneous for the presence of the resistance allele(s). By crossing and backcrossing experiments with a susceptible CM strain, followed by resistance testing of the offspring, an autosomal dominant inheritance of resistance was elucidated. In addition, cross-resistance to CpGV-M and CpGV-S was detected in both strains, CpR5M and CpR5S. To test the hypothesis that the autosomal inheritance of type II resistance was caused by a large interchromosomal rearrangement involving the Z chromosome, making type I resistance appear to be autosomal in these strains; fluorescence *in situ* hybridization with bacterial artificial chromosome probes (BAC-FISH) was used to physically map the Z chromosomes of different CM strains. Conserved synteny of the Z-linked genes in CpR5M and other CM strains rejects this hypothesis and argues for a novel genetic and functional mode of resistance in CM populations with type II resistance.

## Introduction

Baculoviruses are insect pathogenic viruses, which are widely used as biological control agents of insect pests in agriculture and forestry. One of the most important commercially used baculoviruses is the Cydia pomonella granulovirus (CpGV). CpGV belongs to the genus *Betabaculovirus* of the *Baculoviridae* family [1]; its circular dsDNA genome is 123.5 kilobase pairs (kbp) in size and encodes 143 putative open reading frames (ORFs) [2]. CpGV is highly virulent to early larval stages of the codling moth (CM) (*Cydia pomonella*, Lepidoptera: Tortricidae), whereas it is harmless to non-target insects and animals and it has no detrimental impact on the environment [3].

CpGV products have been used since the late 1980s for the control of CM, which is one of the most destructive insect pests in apple, pear and walnut production. Without control, CM can cause severe damage and complete loss of marketable fruits. Pome fruit growers spray formulated viral occlusion bodies (OBs) of CpGV, which are ingested by the CM larvae and cause larval death within four to six days [3].

Although different geographic CpGV isolates have been detected during the last several decades, nearly all worldwide commercially available products contained one CpGV isolate, termed CpGV-M, which was originally discovered in infected CM larvae in Mexico [4]. After a successful use of these CpGV products in organic and integrated pome fruit production, first reports of resistance of CM populations to CpGV appeared in 2005 [5]. Upon further investigation, 38 apple orchards with resistance to CpGV-M based products were identified in Austria (2 orchards), Czech Republic (1), France (3), Germany (22), Italy (6), the Netherlands (2) and Switzerland (2) [6–9]. Some of these CpGV-resistant field populations were collected and reared for genetic and molecular investigations, e.g. the strains CpRR1 (Germany), RGV (France) and CpR-CZ (Czech Republic) [7, 9, 10].

The karyotype of CM consists of 2n = 56 chromosomes, 54 autosomes and two sex chromosomes (W,Z), all of a holokinetic type [11]. CM, like most Lepidoptera, carries a WZ/ZZ sex chromosome system, the females being heterogametic (WZ) and males the homogametic (ZZ) sex [12]. Recently, it was shown by physical mapping that the Z chromosome in CM and other tortricids is the result of a fusion of the ancestral Z chromosome and an autosome corresponding to chromosome 15 in the *Bombyx mori* reference genome, and it is thus a neo-Z chromosome [13]. For CpRR1 it was demonstrated by crossing experiments that CpGV resistance is inherited in a dominant, monogenic and Z-linked mode [7, 14]. Whether the resistance allele of CpRR1 is located on the ancestral part of the neo-Z chromosome or the part corresponding to the *B*. *mori* autosome 15 is unknown.

Based on the following two observations, it was hypothesized that most of the resistant CM populations in Europe follow a similar mechanism and inheritance mode as described for CpRR1 [7,9,10,15]. First, a Z-linked, dominant inheritance was also determined for other, geographically distant CM populations in France and the Czech Republic [9, 15]. Second, most of the resistant CM populations could be successfully controlled by the same resistance-breaking CpGV isolates [10, 16–18]. Phylogenetically, CpGV isolates can be classified into five different genome groups (A-E); the isolate CpGV-M belongs to genome group A [19, 20]. Comparison of the genome sequences of CpGV-M and resistance-breaking isolates belonging to CpGV genome group B-E revealed that all of the latter showed a single common difference to CpGV-M, namely an additional repeat of 2x12 base pairs (bp) within the viral ORF *pe38* in CpGV-M [20]. ORF *pe38* encodes a zinc finger and leucine zipper containing protein and is supposed to be an early transcribed gene in Autographa californica multicapsid nucleopolyhedrovirus (AcMNPV) [21]. These results suggested that the resistance in CpRR1 described so far depends on the particular isolate of the virus [20]. Several of the resistance-breaking CpGV isolates have been already tested in laboratory and field experiments. Most of them demonstrated good efficacy against sensitive and resistant CM strains and some have been eventually registered in different European countries for CM control [10, 17, 18, 22]. With the application of these novel resistance-breaking isolates, successful control of resistant CM field populations was again possible.

However, several apple plantations were recently identified in which even these new resistance-breaking CpGV products failed to control CM sufficiently. One of these CM populations, called NRW-WE, was detected in North-West Germany [23]. This population was not only resistant to isolate CpGV-M of the genome group A, but also to those of genome group C, D and E. Hence, a further type of resistance was proposed. To distinguish these different types of CpGV resistance, it has been proposed to term the Z-linked resistance of CpRR1, specific to CpGV-M, as type I resistance and this novel broader resistance as type II resistance [24]. So far, only CpGV-E2, belonging to genome group B, has been able to break type II resistance [24].

Here, we report the establishment of two genetically homogeneous inbred CM strains, termed CpR5M and CpR5S, which were generated from NRW-WE by a continuous selection procedure on either CpGV-M (genome group A) or CpGV-S (genome group E). Systematic crossing experiments revealed an autosomal dominant inheritance of resistance for both CpR5M and CpR5S; no Z-linkage of resistance was detectable. We further tested the hypothesis that the difference between the Z-linked resistance in CpRR1 and the autosomal resistance in CpR5M could be a consequence of a large-scale rearrangement of the neo-Z chromosome in CpR5M, such as translocation onto an autosome, which would make the type I resistance mechanism appear to be inherited in an autosomal fashion in this strain. Physical mapping of selected marker genes applying fluorescence *in situ* hybridization (FISH) indicated a very similar architecture of the neo-Z chromosome in CpRR1 (type I resistance) and CpR5M (type II resistance) as well as in susceptible CM, rejecting the rearrangement hypothesis.

## Results

### Establishment of genetically homogeneous strains from NRW-WE

**Fig 1:**
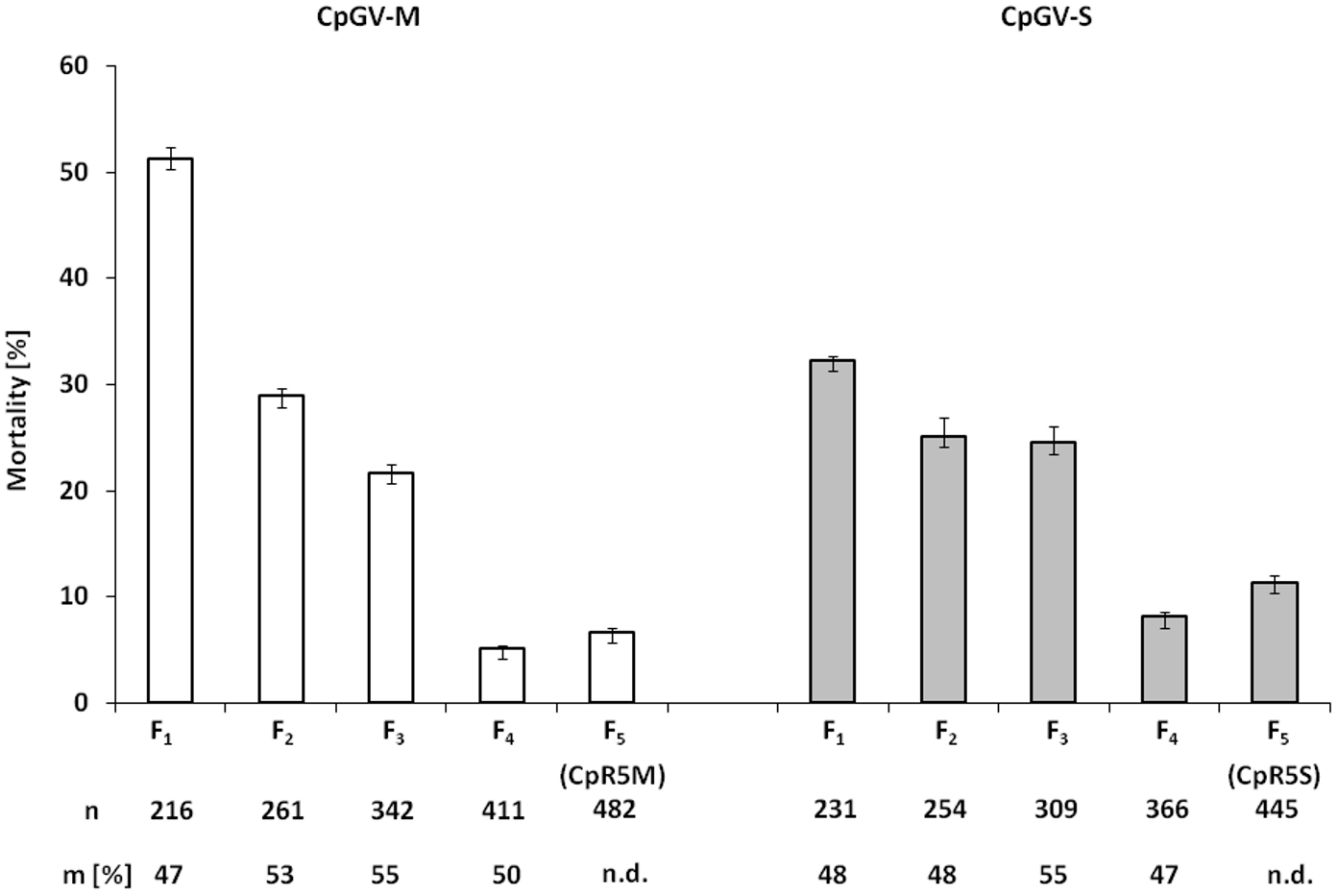
Establishment of genetically homogeneous strains from NRW-WE. Mortality data of first instar larvae during the inbreeding procedure of NRW-WE are shown. Offspring of each generation F_1_-F_5_ were selected on artificial diet containing either CpGV-M (white bars) or CpGV-S (gray bars) at a concentration of 2 x 10^5^ OB/ml. Surviving larvae from each generation were reared to adulthood, followed by subsequent mass cross and selection procedure, resulting in the F_5_ strains CpR5M and CpR5S. The Abbott-corrected mean mortality and standard errors (bars) were determined 16 days post infection [25]. Generation number (F_1_-F_5_), total number of tested individuals (n) and the percentage of surviving male moths (m [%]) are given below the chart, n.d., not determined.

To establish genetically homogeneous CM strains from the field population NRW-WE, five successive inbred crosses (F_1_-F_5_), each followed by an exposure of the larval offspring to selection pressure by either CpGV-M or CpGV-S, were performed. This selection procedure resulted in two resistant CM strains, namely CpR5M (selected on CpGV-M) and CpR5S (selected on CpGV-S) (Fig 1). During five generations of selection on CpGV-M the mortality decreased from 51.3% (F_1_) to 6.7% (F_5_), assessed 16 days post infection (p.i.). When selection was performed on CpGV-S, the virus-induced mortality decreased from 32.3% (F_1_) to 11.4% (F_5_). Thus, the selection procedure caused reduction of mortality by a factor of 7.7 when selected on CpGV-M and by a factor of 2.8 on CpGV-S. The ratio of males among the surviving moths in each of the selected generations was always around 50%, indicating that males and females exhibited an equal survivorship during the selection procedure (Fig 1). Since the last two generations (F_4_ and F_5_) showed a similar mortality, the resistance in generation F_5_ was considered as genetically fixed and the strains were reared without further selection pressure.

### Resistance testing in CpR5M, CpR5S and CpS

**Fig 2:**
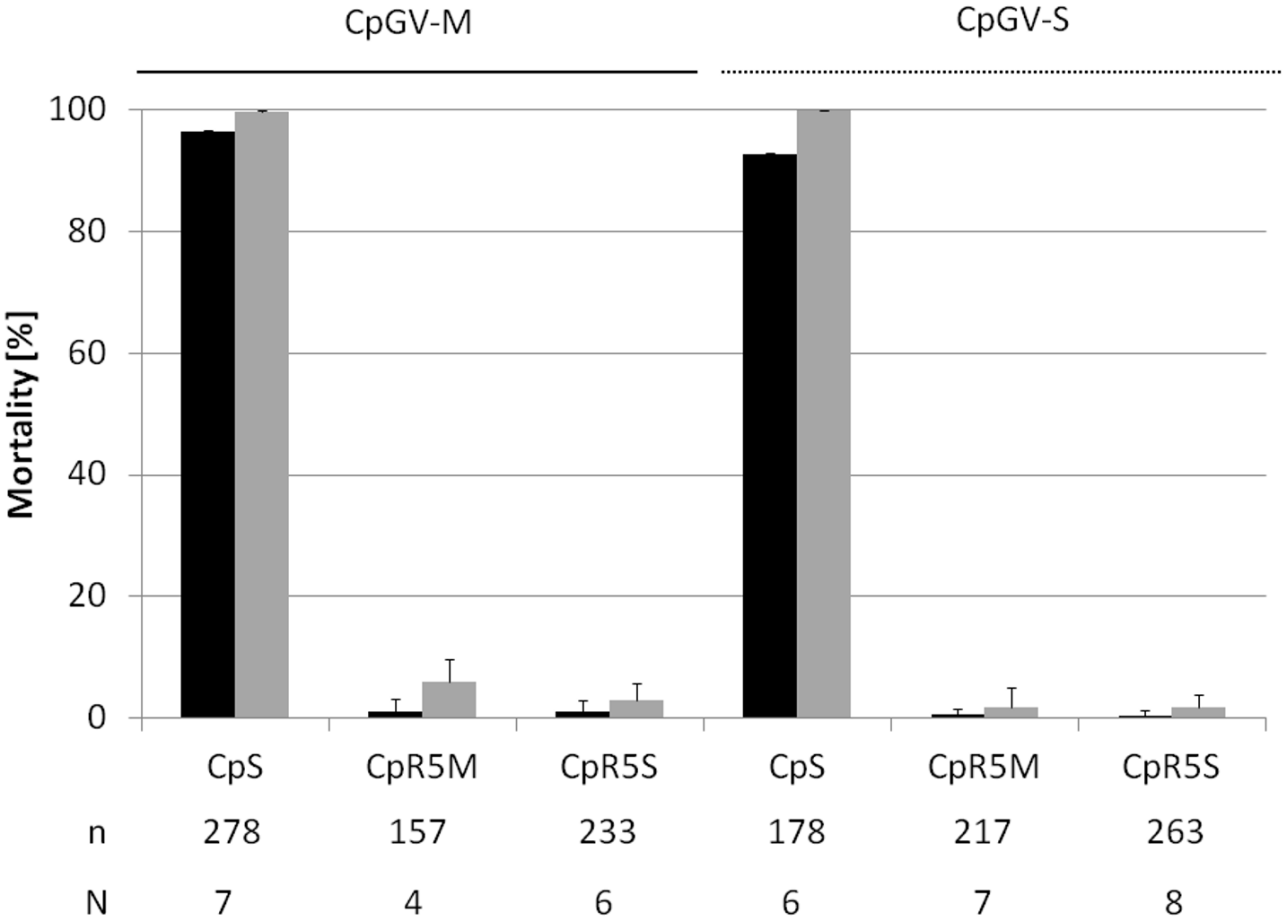
Resistance testing in CpS, CpR5M andCpR5S. Mortality of neonate larvae of CpS, CpR5M or CpR5S tested for resistance on artificial diet containing either CpGV-M (continuous line) or CpGV-S (broken line) at a discriminating concentration of 5.8 x 10^4^ OB/ml. Mean mortality and standard errors after seven (black bars) and 14 days (gray bars) post infection are shown. All mortality data were corrected for control mortality [25]. The total numbers of replicates (N) and tested individuals (n) of the CM strains are given below the chart.

Neonate larvae of the resistant CpR5M and CpR5S as well as the susceptible strain CpS were exposed to the discriminating concentration of 5.8 x 10^4^ OB/ml of CpGV-M and CpGV-S, respectively, to compare the level of resistance. For a susceptible CM colony it is expected that this discriminating concentration of CpGV causes >95% mortality after seven days and >99% mortality after 14 days of virus exposure [7, 20]. For CpS, virus-induced mortality was 96% on CpGV-M and 92% on CpGV-S after seven days and 100% for both viruses after 14 days, as expected (Fig 2). In contrast, the mortality of CpR5M and CpR5S neonates was less than 1% on both CpGV-M and CpGV-S after seven days and less than 6% after 14 days (Fig 2). Thus, both CpR5M and CpR5S strains selected either on CpGV-M (genome group A) or CpGV-S (genome group E) not only showed a highly reduced virus susceptibility compared to CpS but also clear cross-resistance to both CpGV genome groups.

### Crosses and backcrosses of CpR5M or CpR5S with CpS

**Fig 3:**
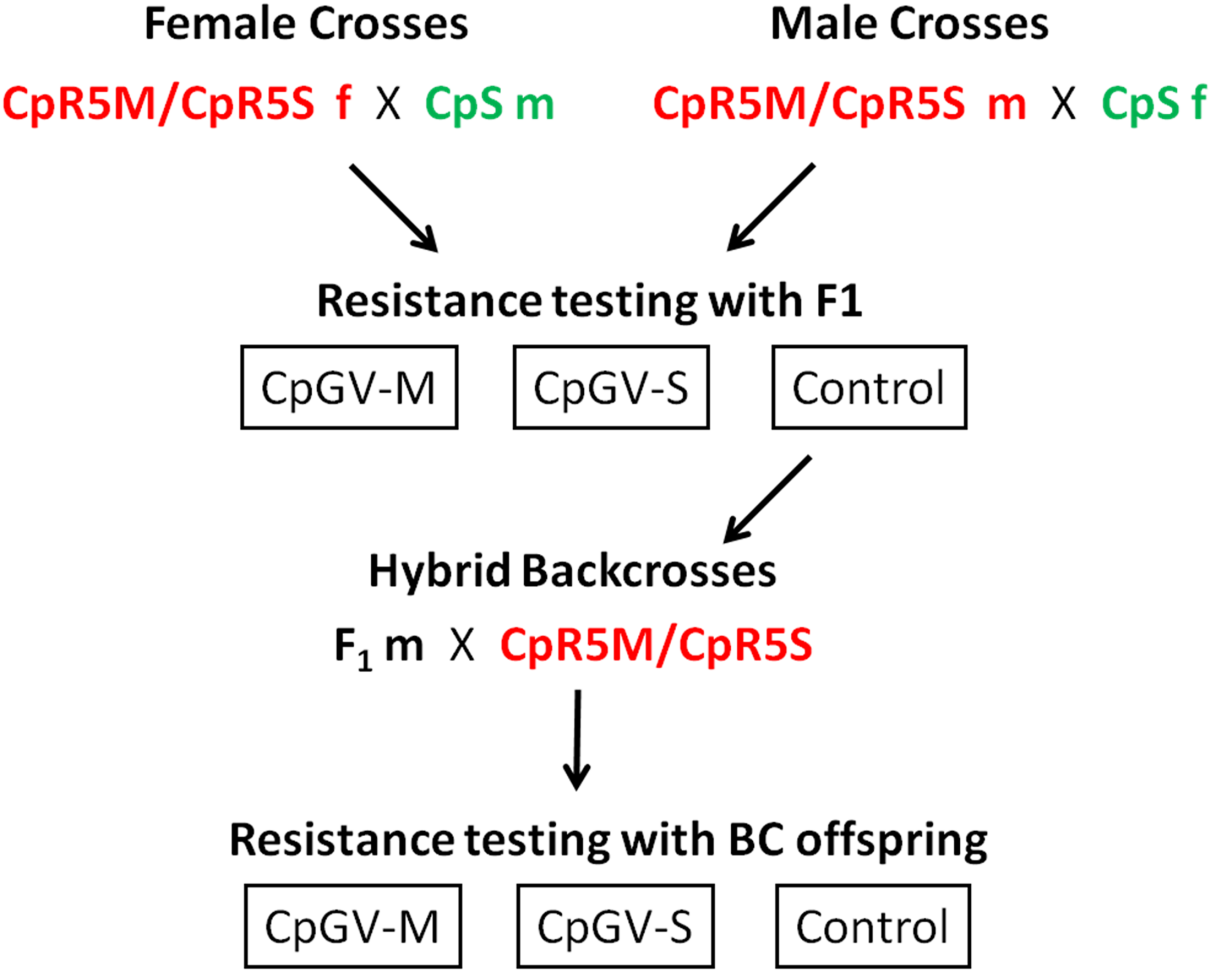
Scheme of hybrid crosses and backcrosses of resistant CpR5M and CpR5S with susceptible CpS. The female crosses started with female moths (f) of CpR5M and CpR5S mated with CpS males (m); and vice-versa in the male crosses. Resulting neonate larvae of the first generation (F_1_) were divided into three cohorts. Two cohorts were tested for resistance with the discriminating concentration 5.8 x 10^4^ OB/ml of CpGV-M and CpGV-S and mortality was observed after seven days. A third cohort of F_1_ offspring was reared as an untreated control on diet without virus until adulthood. For the backcrosses, resulting male moths of the F_1_ control group were mated with females of CpR5M or CpR5S and the backcross (BC) offspring was again tested for resistance using the discriminating concentration.

Hybrid mass crosses and backcrosses with CpS individuals were performed to examine the mode of inheritance of resistance to CpGV-M and CpGV-S in CpR5M and CpR5S (summarized in Fig 3). The autosomal inheritance hypothesis and the Z-linked hypothesis make very different predictions for the mortality rates in certain of these crosses (Table 1). For the female hybrid crosses CpR5Mf x CpSm, mortality in the F_1_ offspring was 0.01 on both CpGV-M and CpGV-S. These results contradicted predictions of Z-linkage of resistance, for which a mortality of 50% would have been expected, and supported the hypothesis of autosomal resistance (0% mortality expected). When F_1_ male moths derived from this hybrid cross were backcrossed with CpR5M females (BC1: F_1_m x CpR5Mf) and their offspring were exposed to CpGV-M and CpGV-S, the mortality was again close to zero, as would be expected for autosomal inheritance (Table 1). The reciprocal male crosses CpR5Mm x CpSf showed mortality rates close to zero on both CpGV-M and CpGV-S, as would be expected for both autosomal and Z-linked inheritance. In the backcross BC2: F_1_m x CpR5Mf, mortality on both CpGV-M and CpGV-S was again zero, also supporting an autosomal mode of inheritance. Analogous hybrid crosses and backcrosses (BC3 and BC4) done with CpR5S yielded very similar results to CpR5M (Table 1). Thus, all crossing and backcrossing experiments fully supported a dominant, autosomal inheritance pattern, while rejecting Z-linkage of resistance.

**Table 1:** Crosses and backcrosses of CpR5M or CpR5S with CpS. Mortality rates of CM larvae determined in resistance tests with neonate larvae seven days post infection (p.i.), exposed to CpGV-M or CpGV-S at a discriminating concentration of 5.8 X 10^4^ OB/ml are shown. The mortality was determined for the offspring of the different strains, CpS, CpR5M, and CpR5S, of the hybrid crosses between CpR5M or CpR5S with CpS, and of the backcrosses. Progeny genotypes are shown for two hypotheses: a single Z-linked, dominant resistance gene (Z) or a single autosomal, dominant resistance gene (A). Abbreviations are BC (backcross), R (resistant allele), S (susceptible allele), F_1_ (offspring of the first generation), f (female), m (male), M (mean), SD (standard deviation), N (number of replicates), and n (total number of tested larvae). Given are the expected (Exp) mortality rates for dominant Z-linked (Z) or autosomal (A) inheritance hypotheses of the F_1_ generation and of the backcrosses. Differences in the expected mortality predicted by Z-linked vs. autosomal inheritance hypotheses are highlighted in gray.

Strain	Crosses	Progeny genotypes, by hypothesis		Mortality rates, 7 days p.i.			
		(Z)	(A)	Exp (Z)	Exp (A)	Observed with CpGV-M M (SD) [N, n]	Observed with CpGV-S M (SD), [N, n]
**CpS**		Z^S^Z^S^, Z^S^W	A^S^A^S^	1.00	1.00	0.97 (0.02) [7, 278]	0.93 (0.03) [6, 178]
**CpR5M**		Z^R^Z^R^, Z^R^W	A^R^A^R^	0.00	0.00	0.01 (0.02) [4, 157]	0.01 (0.01) [7, 217]
**CpR5S**		Z^R^Z^R^, Z^R^W	A^R^A^R^	0.00	0.00	0.01 (0.02) [6, 233]	0.00 (0.01) [8, 263]
**CpR5M**	Female crosses: CpR5Mf X CpSm	Z^R^Z^S^, Z^S^W	A^R^A^S^	0.50	0.00	0.01 (0.02) [3, 234]	0.01 (0.01) [3, 168]
	BC1: F_1_m X CpR5Mf	Z^R^Z^S^, Z^R^Z^R^, Z^S^W, Z^R^W	A^R^A^R^, A^S^A^R^	0.25	0.00	0.01 (0.02) [2, 201]	0.00 (0) [2, 155]
	Male crosses: CpR5Mm X CpSf	Z^R^Z^S^, Z^R^W	A^R^A^S^	0.00	0.00	0.02 (0.03) [4, 425]	0.00 (0) [4, 430]
	BC2: F_1_m X CpR5Mf	Z^R^Z^S^, Z^R^Z^R^, Z^S^W, Z^R^W	A^R^A^R^, A^S^A^R^	0.25	0.00	0.00 (0.01) [2, 343]	0.00 (0) [2, 261]
**CpR5S**	Female crosses: CpR5Sf X CpSm	Z^R^Z^S^, Z^S^W	A^R^A^S^	0.50	0.00	0.04 (0.07) [4, 294]	0.05 (0.11) [4, 327]
	BC3: F_1_m X CpR5Mf	Z^R^Z^S^, Z^R^Z^R^, Z^S^W, Z^R^W	A^R^A^R^, A^S^A^R^	0.25	0.00	0.00 (0) [3, 221]	0.00 (0) [3, 241]
	Male crosses: CpR5Sm X CpSf	Z^R^Z^S^, Z^R^W	A^R^A^S^	0.00	0.00	0.01 (0.01) [4, 298]	0.04 (0.03) [4, 413]
	BC4: F_1_m X CpR5Mf	Z^R^Z^S^, Z^R^Z^R^, Z^S^W, Z^R^W	A^R^A^R^, A^S^A^R^	0.25	0.00	0.00 (0) [3, 267]	0.06 (0.02) [3, 233]

The dominance values were calculated for the mortality rates observed in the female and male hybrid crosses of CpR5M or CpR5S of Table 1. As shown in Table 2 all dominance values ranged between 0.94 and 1.01, and were thus very close to 1 as expected for a dominant trait. Therefore, resistance of CpR5M and CpR5S to CpGV-M or CpGV-S as measured by mortality after seven days was considered to be fully dominant.

**Table 2:** Dominance values and direct test for monogenic inheritance of type II resistance in CpR5M and CpR5S. Dominance values were calculated according to Bourguet [26] using the mortality rates given in Table 1. A direct test of monogenic inheritance for resistance to CpGV-M or CpGV-S [27] was conducted by comparing expected and observed mortality of the backcrosses subjected to CpGV-M and CpGV-S, respectively. Asterisks indicate significant differences from a monogenic model (*χ*^*2*^ *test*, df = 1; *P value < 0.05).

Strain	Crosses	Dominance value		Test for monogenic inheritance			
		CpGV-M	CpGV-S	CpGV-M		CpGV-S	
				*X*^*2*^	*P* value	*X*^*2*^	*P* value
**CpR5M**	Female crosses: CpR5Mf X CpSm	1.00	1.00	-	-	-	-
	BC1: F_1_m X CpR5Mf	-	-	0	1.00	1.57	0.21
	Male crosses: CpR5Mm X CpSf	0.99	1.01	-	-	-	-
	BC2: F_1_m X CpR5Mf			1.72	0.19	1.31	0.25
**CpR5S**	Female crosses: CpR5Sf X CpSm	0.97	0.95	-	-	-	-
	BC3: F_1_m X CpR5Mf	-	-	5.67	0.02*	6.18	0.01*
	Male crosses: CpR5Sm X CpSf	1.00	0.96	-	-	-	-
	BC4: F_1_m X CpR5Mf	-	-	2.70	0.10	19.02	<0.01*

The mortality of the offspring of the backcrosses was also analyzed for monogenic inheritance applying the chi-squared (*χ*^*2*^) test. Whereas the backcrosses of CpR5M were compatible with the assumption for a monogenic inheritance model, significant differences from a monogenic model were observed for CpR5S in three out of four backcrosses (*P* < 0.05) (Table 2).

### BAC-FISH mapping of the neo-Z chromosome of CM strains CpS, CpRR1 and CpR5M

**Fig 4:**
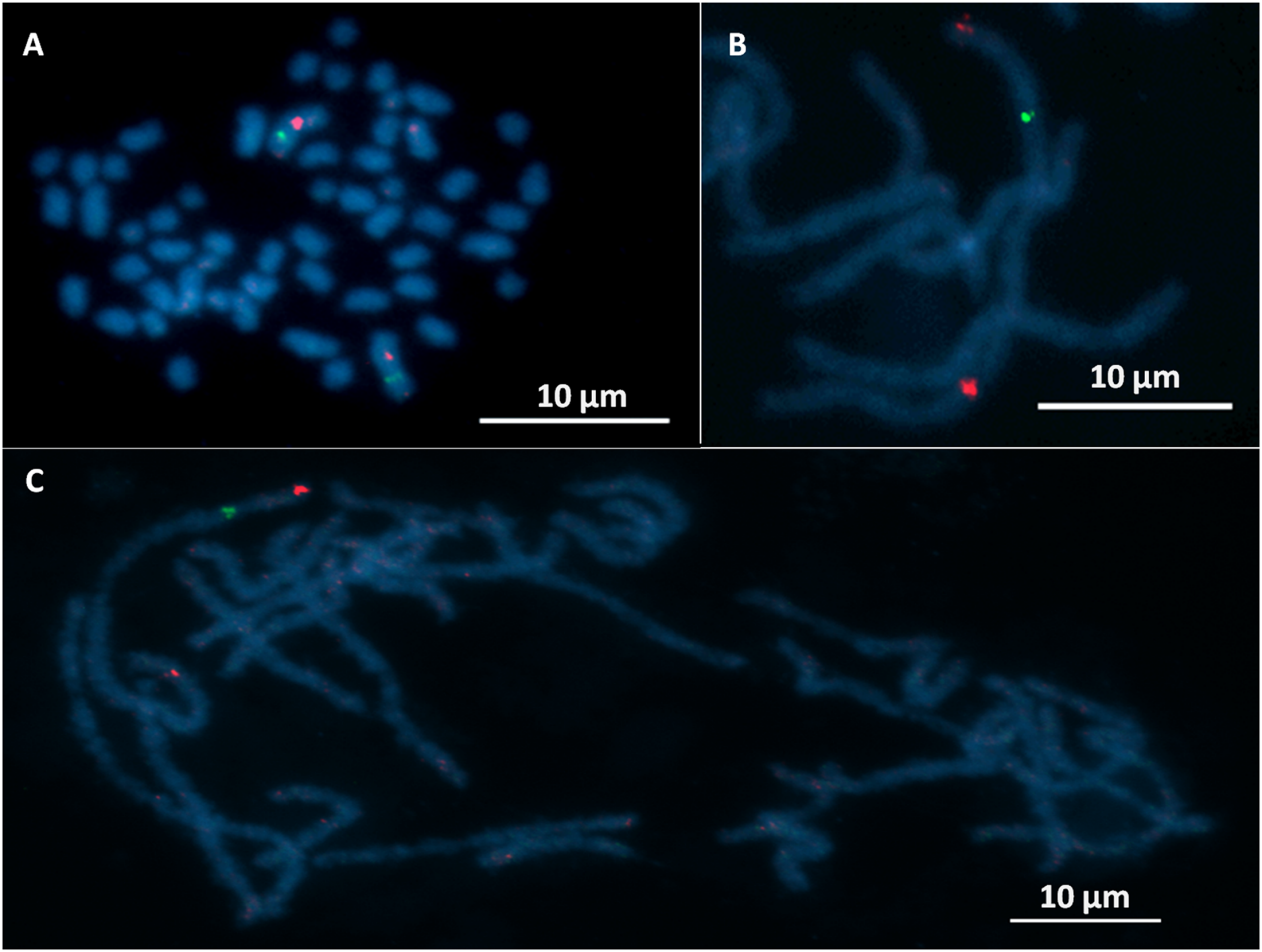
Examples of BAC-FISH mapping of Z-linked genes on the *Cydia pomonella* neo-Z chromosome. Cy3-dUtP-labeled (red hybridization signals) and Fluorescein-12-dUTP-labeled (green) BAC probes were used for BAC-FISH mapping in spread chromosome preparation from testes, counterstained with DAPI (blue). A: CpS-Krym mitotic spermatogonial metaphase showing 2n = 56 chromosomes; two neo-Z chromosomes marked with hybridization signals of BAC probes containing *per* (green) and *nan* (red) genes. B: Detailed view of pachytene ZZ bivalent of CpRR1 with hybridized *ap* (terminal red, arrow), *Tpi* (green) and *Idh-2* (interstitial red) probes. C: CpR5M pachytene complement showing a long ZZ bivalent with hybridized *ap* (terminal red), *ABCF2* (green) and *Notch* (interstitial red, arrow) probes.

**Fig 5:**
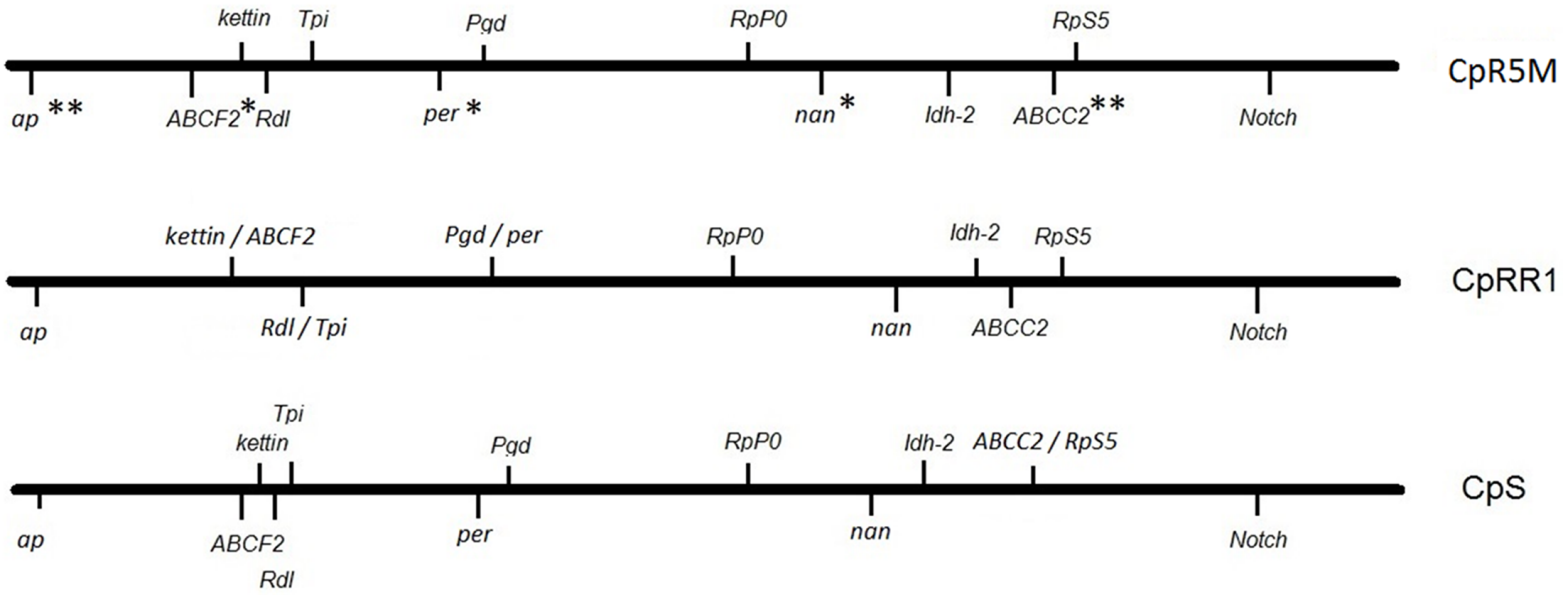
Gene-based schemes of the neo-Z chromosome of CpR5M, CpRR1 and CpS-Krym, integrating all BAC-FISH mapping results. The relative positions of 13 marker gene loci were generated by measuring the physical distance between hybridization signals and the *ap*-labeled chromosome end of at least seven ZZ bivalents of each strain. The measured distances were normalized to the total length of the ZZ bivalent. Genes of CpR5M labeled with one asterisk showed a statistically significant difference in their mean position of the normalized gene loci to CpRR1; genes with two asterisks are significantly different to both CpRR1 and CpS-Krym (ANOVA, Scheffé test, *P* = 0.05).

After the crossing experiments of CpR5M and CpR5S had demonstrated an autosomal inheritance pattern (type II resistance), which differed from the Z-linked inheritance of CpRR1 (type I resistance), we wondered whether a large interchromosomal rearrangement such as a translocation of a part of the neo-Z chromosome onto an autosome in CpR5M could explain the difference. As previously reported [13], the large neo-Z chromosome of *C*. *pomonella* and other tortricids consists of a fusion between an ancestral Z chromosome and an autosome corresponding to the *B*. *mori* reference chromosome 15. If the type I resistance gene were located in the Z chromosome part translocated onto an autosome in CM populations with type II resistance, then the same resistance gene could be responsible for the two resistance types but it would appear to be Z-linked in type I populations and autosomal in type II populations. To examine whether such an interchromosomal rearrangement is involved in type II resistance, the neo-Z chromosomes of CpRR1 and CpR5M were mapped using BAC-FISH, i.e. fluorescence *in situ* hybridization (FISH) with DNA probes prepared from bacterial artificial chromosome (BAC) clones. The BAC clones were selected from the BAC library of *C*. *pomonella* using 13 marker genes located on the neo-Z chromosome of CM [13]. The relative positions of hybridization signals of the BAC probes on the neo-Z chromosome of CpR5M and CpRR1 were measured in at least seven separate and properly spread pachytene nuclei on each slide and compared to those of CpS-Krym, a CM strain that is also susceptible to CpGV [13, 9] (S1 Table). All 13 BAC probes effectively hybridized to the neo-Z chromosome in male pachytene nuclei of the three tested CM strains. The vast majority of analyzed images showed synteny of all marker genes (i.e. location to the same chromosome) and a conserved gene order along the neo-Z chromosome of CpR5M and CpRR1, which were similar to that of susceptible CpS-Krym (Figs 4 and 5). There were only minor local positional differences of *ABCC2* and *per* in CpRR1, but with statistically inconclusive relative distances of the genes (Table 3; Fig 5). Some statistically significant differences of the mean relative distances were detected for *ap* and *ABCF2* genes in CpR5M compared to CpS-Krym and CpRR1; significant differences between CpRR1 and CpR5M were also found for *ABCC2*, *per* and *nan* (ANOVA, Scheffé test, *P* < 0.05) (Table 3; Fig 5). However, BAC-FISH mapping clearly indicated a highly similar overall architecture of the chromosomes of CprR5M, CpRR1 and CpS-Krym and ruled out the possibility of a larger interchromosomal rearrangement of the neo-Z chromosome in the CpR5M strain.

**Table 3:** Overview of the 13 Z-linked genes and summary of statistical analyses of their mapping on the neo-Z chromosome of different codling moth (CM) strains by BAC-FISH. The relative position of hybridization signals of BAC probes to the total length of the neo-Z chromosome in at least seven ZZ bivalents was calculated in each CM strain. Different letters indicate statistical differences in the mean relative position of gene loci (ANOVA, Scheffé test, *P* = 0.05).

Gene name	Symbol	CM strain		
		CpS-Krym	CpRR1	CpR5M
*ABC transporter family C protein ABCC2*	*ABCC2*	AB	A	B
*ABC transporter family F protein ABCF2*	*ABCF2*	A	A	B
*apterous*	*Ap*	A	A	B
*Isocitrate dehydrogenase 2*	*Idh-2*	A	A	A
*kettin*	*kettin*	A	A	A
*nanchung*	*nan*	AB	A	B
*Notch*	*Notch*	A	A	A
*period*	*per*	AB	A	B
*Phosphogluconate dehydrogenase*	*Pgd*	A	A	A
*Resistant to dieldrin*	*Rdl*	A	A	A
*Ribosomal protein P0*	*RpP0*	A	A	A
*Ribosomal protein 55*	*RpS5*	A	A	A
*Triosephosphate isomerase*	*Tpi*	A	A	A

## Discussion

Field resistance to CpGV isolates has become a threat to a successful biological control of codling moth in pome production [7, 28]. A prerequisite for the development of any efficient resistance management strategy is sufficient knowledge of the basic inheritance parameters of the resistance. An atypical broad resistance to different CpGV isolates has recently been described for the field population NRW-WE, hinting at a second type of resistance that differs from the previously reported Z-linked and *pe38*-related type I resistance in CpRR1 [7, 20, 24].

Resistant field populations of insects normally consist of a mixture of homozygous and heterozygous, resistant and susceptible individuals. Genetically homogeneous insect lines are thus a prerequisite for elucidation of inheritance parameters. Two selection methods are typically used for establishing laboratory insect strains with a genetically fixed resistance. One option is continuous inbreeding by single pair crosses followed by resistance testing of the offspring and selecting single families with a uniform resistance response; the other option is mass crossing and selection under virus pressure followed by rearing of the survivors of the selection procedure. Both methods have been successfully applied in studying the inheritance of CpGV resistance [7, 9, 10]. The method of single pair crosses was attempted several times for NRW-WE, however without success because of low progeny numbers and non-uniform response of single families (J.A. Jehle, E. Fritsch, K. Undorf-Spahn, N. Mettenmeyer, A.J. Sauer, unpublished data). Eventually, the establishment of genetically homogeneous strains from the NRW-WE field population was achieved by five rounds of mass crosses each followed by selection under CpGV virus pressure. Although the number of surviving individuals increased during the subsequent selections from F_1_ to F_4_, the sex ratio of the surviving moths was balanced in each generation, pointing already to an inheritance of resistance that is independent from of the neo-Z chromosome.

The ancestral field population NRW-WE still showed mortality of up to 45.6% in resistance tests, when a discriminating concentration of 5.8 x 10^4^ OB/ml of CpGV-M and CpGV-S was applied [24]. Resistance testing at this specific concentration has been previously shown to be a powerful method to differentiate between susceptible and resistant CM individuals, because fully susceptible neonates show generally >95% mortality in a seven-day bioassay, whereas resistant individuals survive this concentration [7, 9]. If neonate CM larvae survive this virus concentration in laboratory tests, it can be assumed that large damage will be caused in the field despite CpGV sprays. Selection of CpR5M and CpR5S resulted in a considerably decreased mortality of less than 6% in any of the resistance tests. Hence, it is obvious that the resistance in NRW-WE was indeed not genetically fixed, whereas CpR5M and CpR5S were genetically homogeneous in terms of CpGV resistance.

Hybrid crosses and backcrosses between the resistant CM strains CpR5M and CpR5S with the susceptible CpS in combination with testing the resistance levels of the offspring were the key to reveal the mode of inheritance of the resistance. Four findings of these crossing experiments are of particular importance:

(i) The observed mortality in all crossing and backcrossing experiments fits perfectly to a dominant, autosomal inheritance of resistance to both viruses CpGV-M and CpGV-S, in both strains CpR5M and CpR5S. These findings clearly explain the previous experimental observations questioning a Z-linked inheritance in NRW-WE [24]. Therefore, it corroborates the previous prediction of a second type (II) of resistance, present in NRW-WE and consequently in CpR5M and CpR5S, which is different from type I resistance in CpRR1.

(ii) Statistical analyses of the hybrid crossing experiments fully supported a dominant mode of inheritance at the given discriminative concentration and test duration. This finding is similar to type I resistance of strain CpRR1 and its ancestor population CpR [7,29]. A dominant inheritance pattern was also described in the French population RGV [10] and the CM strain CpR-CZ from the Czech Republic [9]. The majority of the backcrossing results supported a monogenic mode of inheritance. Nevertheless in three backcrosses monogenic inheritance of resistance to CpGV-S was rejected in favor of an alternative hypothesis. The latter result would imply that more than one gene is responsible for the resistance to CpGV-S but not to CpGV-M. For a more comprehensive picture of the dominance values and the number of alleles involved in the inheritance of type II resistance, a comparison of concentration-mortality response over a range of different virus concentrations needs to be assessed. A multigenic inheritance may explain why we failed to establish a genetically homogeneous line from NRW-WE by single pair crosses.

(iii) As shown by BAC-FISH experiments, the architecture of neo-Z chromosomes in CpR5M (type II resistance) and CpRR1 (type I resistance) was virtually the same and was also highly similar to the susceptible CM strain; all marker genes showed synteny to a single chromosome in all three CM strains. In addition to the Z chromosome-autosome fusion, previous physical mapping of the neo-Z chromosome in tortricids revealed yet another chromosome rearrangement, a translocation of a 0.5- to 2.8-Mb segment of the *B*. *mor*i chromosome 15 from the neo-Z chromosome to an autosome [13]. However, it is unlikely that autosomal resistance in CpR5M could be explained by ancestral polymorphism for this translocation as it originated in the common ancestor of Grapholitini and Olethreutini clades of the subfamily Olethreutinae, to which CM belongs [13]. Since our BAC-FISH experiment included genes more or less evenly distributed along the entire length of the neo-Z chromosome, it is unlikely that a similar translocation of the neo-Z to an autosome would occur in the CM population showing resistance II. Therefore, the autosomal location of the resistance allele in CpR5M (and analogously in CpR5S) cannot be explained by a large scale interchromosome rearrangement, such as translocation of a part of the neo-Z chromosome onto an autosome, which supports our conclusion that the chromosomal location of resistance II is indeed independent from resistance I and not the consequence of a large scale chromosomal rearrangement. Some minor variations in the distance of marker genes to each other might be due to various degree of chromatin condensation, artifacts of chromosome spreading technique or measurement error [30]. Furthermore, we cannot rule out that distances between gene markers were altered by fine scale intrachromosomal rearrangements such as inversions which were shown to be common in Lepidoptera [31] despite the otherwise conserved organization of lepidopteran genomes [32, 33]. Yet none of these would explain why resistance in CpRR1 is Z-linked, whereas it is autosomal in CpR5M, although a translocation of a very small neo-Z chromosome segment involving the resistance gene to an autosome, which escaped our mapping approach, cannot be fully excluded. Unfortunately, a high-resolution map that would allow a fine scale inter-strain comparison is not available in CM.

(iv) Selection of NRW-WE either on CpGV-M (genome group A) or CpGV-S (genome group E) resulted in two strains, CpR5M and CpR5S, which were both cross-resistant to CpGV-M and CpGV-S. This observation would argue in the first instance for a functional cross-resistance mechanism against both CpGV-M and CpGV-S that must be different from the mechanism of type I resistance. As shown for CpRR1, type I resistance is targeted against a 24 bp repeat in *pe38* of genome group A CpGVs, such as CpGV-M, but not in other CpGVs [20]. Since CpGV-S also lacks this repeat in *pe38*, this locus cannot be the major target of type II resistance [20, 24]. Therefore, a novel resistance mechanism must be involved in type II resistance, which would also explain its different (i.e. autosomal) mode of inheritance. Although our experiments provide clear evidence for a dominant and autosomal (cross) resistance to CpGV-M and CpGV-S in CpR5M and CpR5S, they do not fully explain the results of hybrid crosses obtained with the parental CM strain NRW-WE [24]. There, single pair hybrid crosses between individuals of NRW-WE and susceptible CpS resulted in a highly heterogeneous response of different hybrid families, with highly variable mortality rates in different families, not supporting a simple model of cross resistance to CpGV-M and CpGV-S. Hence, additional factors or phenotypic plasticity may be involved in the resistance observed in the field populations, which may have been lost during the inbreeding and selection procedure to generate genetically homogeneous strains.

The discovery of type II resistance with a mechanism and inheritance that are different to those of type I resistance underscores the high capacity of adaptation of CM to CpGV isolates, posing a continuous threat to the successful use of CpGV products in organic and integrated pome fruit production. Since resistance to CpGV is a widespread phenomenon in Europe, it is important to identify the specific resistance mechanism in these populations to avoid control failure when novel resistance-breaking CpGV products are applied. The existence of a second type of CpGV resistance necessitates particular attention of research, extension services and commercial CpGV producers when developing and optimizing resistance management strategies for CpGV.

## Materials and methods

### Insects

The CpS strain of *C*. *pomonella* is susceptible to all CpGV isolates and has been reared at the Julius Kühn Institut (JKI) in Darmstadt (Germany) for many years [7]. The CM strain CpS-Krym (= Krym-61) shows the same susceptibility to all CpGV isolates as CpS and was used as a reference in the fluorescence *in situ* hybridization (FISH) experiments [9, 11, 13]. CpRR1 carries type I resistance against CpGV-M and arose from the resistant field population CpR (BW-FI-03, ‘Sudbaden’) by single pair crosses [7]. The resistant field population NRW-WE descended from an apple orchard in North Rhine-Westphalia (Germany) and had a reduced susceptibility to CpGV-M and CpGV-S [7, 24]. The CM inbred lines CpR5M and CpR5S were selected form NRW-WE in the course of this study (see below). All CM strains were reared under laboratory conditions at 26°C with 16/8 h light/dark photoperiod and 60% relative humidity on modified diet of Ivaldi-Sender [34].

### Viruses

Different isolates of Cydia pomonella granulovirus (CpGV) were used in this study. CpGV-M (genome group A), the so-called “Mexican isolate” [4], was prepared from batch TPCpGVBTPS_02 [20]. The isolate CpGV-S (genome group E) derived from the commercial product Virosoft (BioTEPP Inc., Canada) and was propagated in CpS larvae. Purification of virus occlusion bodies (OB) was done as described before [35]; all samples were kept at -20°C. Quantification of virus stocks was performed by OB counting in dark-field optics of a light microscope (Leica DMRBE) with the Pertoff-Hauser counting chamber (depth 0.02 mm).

### Resistance testing

Resistance testing was performed as previously described [36] applying the diet incorporation method [34]. Purified OB of CpGV-M and CpGV-S were mixed into the modified diet of Ivaldi-Sender [34] at a final discriminating concentration of 5.8 x 10^4^ OB/ml. This concentration caused >95% mortality to neonate CpS larvae in seven day bioassays [7]. Neonate larvae of NRW-WE, CpR5M and CpR5S were placed on the diet and mortality was determined one, seven and 14 days post infection (p.i.). Only larvae surviving day were considered to be introduced into the test. The observed virus-induced mortality was corrected by the mortality of the untreated control group [25].

### Establishing the virus selected strains CpR5M and CpR5S

For establishing genetically homogeneous lines from the field population NRW-WE, mass crosses under CpGV selective pressure were performed as described elsewhere [9, 10]. Emerged first instar larvae of the mass reared NRW-WE were selected on modified Ivaldi-Sender diet containing either CpGV-M or CpGV-S at a selective concentration of 2 x 10^5^ OB/ml. Larval mortality was recorded 16 days p.i. Larvae surviving virus treatment were reared until pupation and the sex of the pupae was noted by determining the number of their abdominal segments [29]. Emerging adults were again mass-crossed for the second round and the larval offspring was again subjected to the selection process. This procedure was repeated for five generations. The strain CpR5M was the outcome of the successive mass selection on CpGV-M, whereas CpR5S originated from continuous selection on CpGV-S. These two strains were reared separately under laboratory conditions without further exposure to CpGV.

### Reciprocal crosses and backcrosses

To analyze the mode of inheritance of the resistance allele(s) in the selected strains CpR5M and CpR5S, reciprocal crosses between these two strains and CpS were performed. Resulting pupae were again separated by sex, and further used in two genetic hybrid crosses (Fig 3); in the female crosses, emerging resistant female moths from either CpR5M or CpR5S were mated with males from the sensitive CpS strain. In the hybrid male crosses, resistant male moths were mass crossed with CpS females. Each cross consisted of eight to ten moths with a ratio of 1:1 males to females. Eggs were collected every second day. Neonate larvae of the first generation (F_1_) were divided into three cohorts and subjected to artificial diet containing either (i) CpGV-M, or (ii) CpGV-S, each at a discriminating concentration of 5.8 x 10^4^ OB/ml, or (iii) virus free diet as untreated control. Mortality in all three cohorts was scored after one and seven days. The larvae of the control were further reared to adulthood and used for backcrossing experiments (see below).

For backcrosses, hybrid F_1_ male moths were crossed with females of the parental strains, CpR5M or CpR5S, respectively. Each backcross consisted of eight to ten F_1_ male moths and an equal number of female moths. The neonate offspring were tested for resistance on CpGV-M or CpGV-S at a discriminating concentration of 5.8 x 10^4^ OB/ml after seven days or were reared on untreated diet as described above. Hybrid crosses and backcrosses were independently repeated three to five times.

### BAC-FISH mapping

To compare the genetic architecture of the neo-Z chromosome of CpR5M and CpRR1 (with the susceptible CpS-Krym as a reference) fluorescence *in situ* hybridization (FISH) with probes prepared from DNA of *C*. *pomonella* bacterial artificial chromosome (BAC) clones, selected from the *C*. *pomonella* BAC library was used, as described in Nguyen et al. [13]. The BAC clones contained in total 13 genes located on the Z chromosome of *C*. *pomonella* [13]. Three insecticide resistance genes (*ABCC2*, *ABCF2* and *Rdl*), three enzyme-coding genes (*Tpi*, *Idh-2* and *Pgd*) and seven genes encoding non-enzymatic proteins (*ap*, *Notch*, *kettin*, *RpS5*, *nan*, *per* and *RpP0*) were chosen as marker genes (Table 3). Meiotic chromosomes were prepared from gonads of late fourth instar male larvae of the three CM strains, CpS-Krym, CpRR1 and CpR5M. Chromosomes were spread on slides using a heating plate and meiotic stage was checked under a phase contrast microscope [11]. BAC DNA was extracted using the Qiagen Plasmid Midi Kit (Qiagen, Düsseldorf, Germany) and labeled by Cy3-dUTP (GE Healthcare, Buckinghamshire, UK) or ChromaTide Fluorescein-12-dUTP (Invitrogen, Paisley, UK) using a Nick Translation Kit (Abbott Molecular, Des Plaines, IL, USA). Labeled BAC probes were hybridized to chromosome preparations using two-color BAC-FISH as described in [13]. BAC-FISH preparations were counterstained with DAPI and observed in a Zeiss Axioplan 2 microscope (Carl Zeiss, Jena, Germany) equipped with appropriate fluorescence filter sets. In total, more than 1100 images of clearly visible and complete pachytene neo-ZZ bivalents were recorded using an Olympus CCD monochrome camera XM10 equipped with cellSens 1.9 digital imaging software (Olympus Europa Holding, Hamburg, Germany) pseudocolored and superimposed with Adobe Photoshop CS3. Due to the absence of the primary restriction, the centromere, in holokinetic chromosomes of Lepidoptera [37, 38], the *apterous (ap)* gene was selected as the common marker for calculating the relative distance of different gene loci along the neo-Z chromosome. By probing different pairs of genes while keeping *ap* as a reference, six different BAC-FISH experiments were performed to complete the mapping of all 13 marker genes in each CM strain. The images were analyzed using the freeware ImageJ (National Institutes of Health). The relative position of hybridization signals was measured three times and normalized for the whole length of the neo-Z chromosome. The tip of the neo-Z chromosome at the *ap* end was defined as the zero point.

### Statistical methods

Dominance of resistance (*D*_x_) was calculated for the hybrid crosses [26]. *D*_X_ = (*x*_*RS*_−*x*_*SS*_)/(*x*_*RR*_−*x*_*SS*_), where *x*_*SS*_, *x*_*RS*_ and *x*_*RR*_ were the observed mortality rate of the susceptible homozygous, heterozygous and resistant homozygous individuals, respectively. Values of *D*_*X*_ ranged from 0 to 1. D values close to 0 were considered to represent a completely recessive inheritance, whereas values close to 1 represent a completely dominant resistance [26].

Statistical analyses of backcrosses were performed with a *χ*^*2*^ test of fit (df = 1) between the expected and observed backcross mortality at the discriminating concentration for monogenetic inheritance [27]. The expected number of deaths was calculated as *n*_*i*_*M*_*i*_, where *n*_*i*_ is the mortality scored at the discriminating concentration *i* and *M*_*i*_ = 0.5 x (*W*_*RS*_ + *W*_*SS*_) or *M*_*i*_ = 0.5 x (*W*_*RS*_ + *W*_*RR*_) depending on the type of backcross and the CM strain. *W*_*RS*_, *W*_*RR*_ and *W*_*SS*_ are the mortality of the tested hybrid F_1_ (RS) and homozygous parental (RR) or (SS) lines at the discriminating concentration *i*.

Differences of the means in positions of marker genes located on the neo-Z chromosome in the BAC-FISH analyses were statistically examined by ANOVA and the Scheffé test of the Agricolae Package (software R studio; edition 2.3.4.4.). *P* < 0.05 was applied as the significance level.

## Supporting information

S1 Table Means of the relative position of 13 marker genes located on the neo-Z chromosome of codling moth strains CpS-Krym, CpRR1 and CpR5M. The relative positions of the marker gene loci were generated by measuring the physical distance between hybridization signals and the *ap*-labeled chromosome end of neo-ZZ bivalents in the strains. The measured distances were normalized to the total length of the neo-ZZ bivalent; given are the total number of neo-ZZ bivalents that were measured (N) and standard deviation (SD).
(DOCX)
