# Supplementary material for: Novel resistance to Cydia pomonella granulovirus (CpGV) in codling moth shows autosomal and dominant inheritance and confers cross-resistance to different CpGV genome groups
Source: PLoS One. 2017 Jun 22;12(6):e0179157. doi: 10.1371/journal.pone.0179157 (PMC5480857; doi:10.1371/journal.pone.0179157)
Supplement: S1 Table — The relative positions of the marker gene loci were generated by measuring the physical distance between hybridization signals and the ap-labeled chromosome end of neo-ZZ bivalents in the strains. The measured distances were normalized to the total length of the neo-ZZ bivalent; given are the total number of neo-ZZ bivalents that were measured (N) and standard deviation (SD). (DOCX) [file pone.0179157.s001.docx]

**S2 Table.** **Means of the relative position of 13 marker genes located on the neo-Z chromosome of codling moth strains CpS-Krym, CpRR1 and CpR5M.** The relative positions of the marker gene loci were generated by measuring the physical distance between hybridization signals and the *ap*-labeled chromosome end of neo-ZZ bivalents in the strains. The measured distances were normalized to the total length of the neo-ZZ bivalent; given are the total number of neo-ZZ bivalents that were measured (N) and standard deviation (SD).

| **Marker gene** | **CpS-Krym** | | | **CpRR1** | | | **CpR5M** | | |
| --- | --- | --- | --- | --- | --- | --- | --- | --- | --- |
|  | **N** | **mean** | **SD** | **N** | **mean** | **SD** | **N** | **mean** | **SD** |
| ***ABCC2*** | 9 | 0.731 | 0.023 | 13 | 0.722 | 0.043 | 15 | 0.754 | 0.024 |
| ***ABCF2*** | 11 | 0.163 | 0.039 | 9 | 0.162 | 0.010 | 15 | 0.131 | 0.014 |
| ***ap*** | 68 | 0.018 | 0.005 | 67 | 0.019 | 0.005 | 75 | 0.015 | 0.004 |
| ***Idh-2*** | 7 | 0.656 | 0.093 | 12 | 0.697 | 0.027 | 10 | 0.679 | 0.055 |
| ***kettin*** | 12 | 0.176 | 0.021 | 9 | 0.160 | 0.034 | 12 | 0.168 | 0.017 |
| ***nan*** | 17 | 0.618 | 0.032 | 11 | 0.639 | 0.024 | 11 | 0.587 | 0.040 |
| ***notch*** | 11 | 0.898 | 0.037 | 9 | 0.901 | 0.030 | 15 | 0.911 | 0.014 |
| ***per*** | 17 | 0.334 | 0.034 | 11 | 0.353 | 0.026 | 11 | 0.310 | 0.029 |
| ***Pgd*** | 12 | 0.356 | 0.031 | 13 | 0.345 | 0.045 | 12 | 0.343 | 0.048 |
| ***Rdl*** | 9 | 0.188 | 0.013 | 13 | 0.205 | 0.289 | 15 | 0.186 | 0.019 |
| ***PpP0*** | 12 | 0.529 | 0.050 | 13 | 0.521 | 0.058 | 12 | 0.533 | 0.064 |
| ***RpS5*** | 12 | 0.740 | 0.040 | 9 | 0.760 | 0.033 | 12 | 0.770 | 0.031 |
| ***Tpi*** | 7 | 0.200 | 0.038 | 12 | 0.216 | 0.025 | 10 | 0.219 | 0.032 |
